# Short-term decongestion and clinical benefit in acute heart failure: a systematic review and trial-level meta-regression

**DOI:** 10.3389/fcvm.2026.1942130

**Published:** 2026-10-30

**Authors:** Huiming Wang, Hang Fang, Mengqi Wu, Qiang Chen, Anyi Fu, Mengdan Xu

**Affiliations:** Department of Cardiology, the Quzhou Affiliated Hospital of Wenzhou Medical University, Quzhou People’s Hospital, Quzhou, China

**Keywords:** acute heart failure, body weight, decongestion, meta-regression, natriuresis, surrogate endpoint

## Abstract

**Background:**

Body weight, urine output, urinary sodium, and ultrasound findings are frequently used as early trial endpoints in acute heart failure (AHF). However, patient-level prognostic associations with residual congestion do not establish the fact that treatment-induced marker improvement predicts fewer deaths or heart failure events.

**Objectives:**

The objectives of this work are to pair short-term decongestion and subsequent clinical treatment effects within the same randomized comparisons, explore the trial-level association for body weight, and qualitatively assess non-weight markers.

**Methods:**

PubMed/MEDLINE, OpenAlex, and ClinicalTrials.gov databases were searched through 18 July 2026. Eligible randomized adult AHF trials reported an objective decongestion effect and ≥30-day mortality, heart failure events, or rehospitalization. The amended primary marker was weight change at 24–96 h (original window, 48–96 h); clinical effects were log hazard or risk ratios. An errors-in-variables meta-regression model used 10,000 trial cluster bootstrap fits and trial-level jackknife intervals.

**Results:**

Twenty-four trials (27 reports) were accepted for qualitative synthesis; the primary analysis included 12 comparisons from only 10 independent trials. Endpoints, follow-up (30–302 days), effect measures, and mechanisms were heterogeneous. Each additional 1-kg between-group difference in weight loss corresponded to a clinical effect ratio of 0.74 (percentile 95% interval, 0.56–1.26; jackknife, 0.49–1.11). The pharmacological-only estimate was 1.04. Evidence for urine output, urinary sodium, and ultrasound was also inconsistent.

**Conclusions:**

Short-term weight loss showed a directional but non-robust trial-level association with clinical benefit. The randomized trial-level evidence evaluated here is insufficient to validate short-term weight change as a surrogate for post-discharge clinical outcomes in AHF.

## Introduction

Congestion is the dominant treatable pathophysiological state in patients hospitalized with acute heart failure (AHF). Elevated pulmonary filling pressure produces dyspnea, while systemic venous congestion contributes to kidney dysfunction, hepatic congestion, bowel-wall edema, and diuretic resistance. Contemporary guidelines therefore place intravenous loop diuretics and repeated assessment of decongestive response at the center of inpatient management (1). The unresolved clinical problem is rarely whether a congested patient should receive diuretics. It is how to decide whether decongestion is sufficient and safe, and whether an improvement observed during admission is likely to translate into benefit after discharge.

Body weight is inexpensive, intuitive, and repeatedly measurable, and it has consequently become a common efficacy endpoint in AHF trials. In DOSE, an intensified loop diuretic strategy produced greater fluid and weight loss, but did not significantly reduce clinical events through 60 days (2). A subsequent analysis of the same trial population showed that patient-level changes in weight or fluid balance did not map simply onto symptom relief and post-discharge outcomes (3). This distinction is fundamental: A marker may be prognostic because patients with persistent congestion are sicker, yet fail as a treatment-effect surrogate if interventions that improve the marker do not reliably improve the clinical endpoint.

Decongestion measures also represent different physiological processes. Body weight is influenced by baseline measurement, intake, bowel movements, and changes unrelated to extracellular fluid. Urine volume primarily reflects water excretion; vasopressin antagonism, for example, can produce substantial aquaresis without proportional sodium removal. In the EVEREST clinical status trials, tolvaptan improved early weight and selected congestion symptoms (4), whereas the outcome trial showed no reduction in mortality or heart failure hospitalization (HFH) (5). Urinary sodium and cumulative sodium excretion may more directly reflect natriuretic response, but are themselves sensitive to sampling time, kidney function, prior diuretic exposure, urine volume, and the distinction between concentration and total excretion.

Ultrafiltration trials further illustrate the unstable link between fluid removal and benefit. UNLOAD reported greater weight loss at 48 h and fewer heart failure rehospitalizations through 90 days (6). In contrast, CARRESS-HF enrolled patients with cardiorenal syndrome and found similar weight loss, worse renal function, more serious adverse events, and no clinical advantage through 60 days (7). A single numerical measure of “more decongestion” can therefore conceal consequences of treatment mechanism, removal rate, vascular refilling, renal perfusion, and patient selection.

Previous evidence syntheses have generally compared the average effects of diuretic strategies on weight, urine output, renal outcomes, or clinical events. A recent network meta-analysis observed that congestion scores and clinical outcomes were reported in only about half of eligible AHF diuretic trials and that strategies producing more weight loss did not yield a consistent clinical advantage (8). Reviews of natriuresis-guided treatment address whether the guidance strategy improves outcomes, rather than whether the randomized effect on natriuresis predicts the randomized effect on clinical events (9). We found no synthesis that systematically paired the short-term objective decongestion effect and the subsequent clinical effect from the same randomized comparison and directly evaluated their trial-level relation.

We therefore asked an exploratory trial design question with direct clinical relevance: when an intervention produces more short-term decongestion than its randomized comparator, is that advantage accompanied by fewer deaths, heart failure events, or rehospitalizations? Body-weight change was selected as the primary marker because it was the most consistently reported measure. Urine output, natriuresis, natriuretic peptides, and ultrasound-defined residual congestion were evaluated in a separate structured qualitative synthesis. Our purpose was not to dismiss the clinical utility of these measures, but to explore the trial-level treatment-effect association without claiming formal surrogate validation.

## Methods

### Protocol and reporting

A protocol was finalized and saved before statistical analysis. Reporting followed the PRISMA 2020 guidelines (10). The original protocol specified a 48–96-h primary marker window. Before viewing meta-regression results, extraction of pivotal trials showed that 24-h weight was a consistently available early measure in EVEREST and other studies. The primary window was therefore amended to 24–96 h, while the original 48–96-h window was retained for a main-manuscript sensitivity analysis (Table 1). Local protocol files, the amendment, filesystem times, and SHA-256 hashes document the supplied files but are not equivalent to prospective public registration. The review was not prospectively registered in PROSPERO.

**Table 1:** Meta-regression and sensitivity analyses.

Analysis	Comparisons/trials	Clinical effect ratio per kg (bootstrap 95% interval)	Trial-jackknife 95% interval	Residual *τ*	Interpretation
Primary 24–96 h	12/10	0.74 (0.56–1.26)	0.49–1.11	0.000016	Exploratory primary association
Original 48–96 h	10/8	0.74 (0.48–1.36)	0.42–1.29	0.000017	Original protocol window
One comparison per trial	10/10	0.69 (0.53–1.10)	0.52–0.91	0.000011	Shared-participant check
Pharmacological only	9/7	1.04 (0.55–1.55)	0.32–3.35	0.000013	Mechanism restriction
HF-event endpoint family	5/5	0.64 (0.57–0.99)	0.34–1.21	0.000057	More homogeneous endpoint family
60–90 day clinical follow-up	8/6	0.75 (0.46–1.45)	0.39–1.44	0.000024	More homogeneous follow-up
HR estimates only	5/4	0.68 (unstable)	0.18–2.62	0.000048	Only four clusters; unstable bootstrap
Count-based RR estimates only	6/5	0.82 (0.47–1.11)	0.52–1.29	0.000151	Effect measure check
Exclude high risk of bias	11/9	0.78 (0.54–1.37)	0.34–1.76	0.000080	Removes Early UF only
Low risk of bias only	9/7	1.04 (0.55–1.55)	0.32–3.35	0.000013	Identical to pharmacological-only set
Exclude mortality-only	10/9	0.64 (0.51–1.03)	0.51–0.81	0.000010	Exploratory outcome selection analysis
Expanded timing set	14/12	0.70 (0.37–0.96)	0.50–0.97	0.000067	Exploratory; mixed marker timing
Exclude KM-risk approximation	11/9	0.73 (0.59–1.27)	0.50–1.07	0.000008	*Post hoc* source audit check
Exclude denominator/interval approximations	7/6	0.74 (0.59–1.30)	0.44–1.23	0.000006	*Post hoc* source audit check
Inflate uncertain weight SEs by25%	12/10	0.74 (0.54–1.32)	0.50–1.11	0.000007	*Post hoc* source audit check
Retain original CARRESS/TACTICS time points	12/10	0.75 (0.58–1.26)	0.50–1.12	0.000005	*Post hoc* source audit check

Clinical effect ratio = exp(*β*); values <1 indicate that comparisons with greater weight loss tended to have more favorable relative clinical effects. Primary percentile intervals used 10,000 successful trial cluster bootstrap fits; sensitivity set percentile intervals used 1,000 successful fits and are accompanied by trial-jackknife intervals. Boundary-near τ was weakly identified and does not establish homogeneity; the approximate likelihood support upper bound was 0.130. Endpoint, follow-up, effect measure, and risk-of-bias restrictions are exploratory because they contain few trials. The HR-only percentile interval is marked unstable because of extreme bootstrap slopes; no numerical lower limit suggesting high precision is inferred from that distribution. The four source-approximation/timing checks are *post hoc*.

### Eligibility criteria

We included randomized controlled trials that (1) enrolled adults aged ≥18 years hospitalized with *de novo* AHF or acute worsening of chronic HF and clinical or imaging evidence of congestion; (2) compared a pharmacological, ultrafiltration, monitoring-guided, or other active decongestive strategy with a randomized control; (3) reported a between-group effect on at least one objective decongestion measure; and (4) reported all-cause or cardiovascular death, HFH, an urgent heart failure visit, or a composite containing these events after at least 30 days. We excluded single-arm and observational studies, trials confined to stable chronic heart failure, studies reporting only symptoms or length of stay, and reports in which decongestion and clinical effects could not be paired within the same randomized comparison.

The amended primary marker was the between-group difference in weight change at 24–96 h. Additional markers were cumulative urine output, urinary sodium or total sodium excretion, N-terminal pro-B-type natriuretic peptide (NT-proBNP) or B-type natriuretic peptide (BNP), hemoconcentration, lung B-lines, and inferior vena cava indices. When several marker times had recoverable treatment effects and uncertainty, we selected the time closest to 72 h within the eligible window. The clinical outcome hierarchy was death or heart failure event composites, heart failure rehospitalization, all-cause rehospitalization, and all-cause death. Symptoms, kidney function changes, and length of stay were not considered patient-important clinical outcomes for the primary pairing.

### Information sources and study selection

PubMed/MEDLINE, OpenAlex, and ClinicalTrials.gov were searched through 18 July 2026, using the source-specific date limits provided in Appendix 1. Search concepts covered acute or decompensated heart failure; decongestion, diuresis, natriuresis, weight, urine output, and ultrafiltration; randomized trials; specific drug classes; and guidance strategies. Backward and forward citation chasing was performed from included trials, relevant systematic reviews, and major guidelines. The executed electronic strategies are provided in Appendix 1. No language restriction was applied at the search stage, and no report remained awaiting classification because it could not be translated. Embase and CENTRAL were unavailable and were not searched. No dedicated conference abstract database was systematically searched; conference-only records encountered through citation chasing or registry searches were screened. No non-public author-supplied efficacy dataset entered the quantitative model.

Records were deduplicated using PMID, DOI, registry identifier, and normalized title. At title/abstract stage, prespecified high-sensitivity keyword rules assisted prioritization and screening. Every record entering the candidate set and every full-text report was then individually verified; automated rules did not make full-text inclusion decisions. One investigator performed title/abstract screening, full-text assessment, data extraction, and risk-of-bias assessment, followed by a same-investigator source verification pass. There was no independent disagreement-resolution process or second-reviewer sample check; these procedures were not independently duplicated.

### Data extraction and effect construction

Trial design, sample size, intervention, comparator, decongestion measure and timing, clinical endpoint, follow-up, and treatment-effect estimates were extracted from primary reports, companion analyses, and public registries. Continuous marker effects were oriented so that positive values indicated greater decongestion with the intervention. Weight was expressed in kilograms (1 lb = 0.45359237 kg). Reported between-group estimates and uncertainty were preferred; otherwise, standard errors were calculated from group means, SDs, and outcome-specific sample sizes where available. CLOROTIC used its adjusted weight contrast with a normal-approximation standard error (SE) recovered from the reported interval. DOSE and SECRET of CHF lacked a verified outcome-specific weight denominator; reported arm or treated sample sizes were used as explicit approximations. Source verification of included trials on 7 September 2026 recovered matched registry weight data for CARRESS-HF at 72 h, ATHENA-HF at 96 h or earlier discharge, and TACTICS-HF at 72 h (11–13). The CARRESS-HF and TACTICS-HF time points were corrected to apply the existing closest-to-72-h rule; retaining the previous 96- and 48-h time points was examined separately. This targeted verification did not constitute a new systematic search. Weight protocols were accepted as reported. Most reports did not provide enough detail to verify a common calibrated scale, fixed time of day, standardized clothing, fasting state, or pre/post-void conditions. CARRESS-HF was an exception: It specified morning weighing before breakfast using the same scale and standardized clothing (7).

Unadjusted or baseline-adjusted hazard ratios were preferred; adjustment for post-randomization decongestion or renal variables was avoided. Accordingly, the expanded Palazzuoli comparison used the reported univariate treatment hazard ratio (HR). Reported risk ratios or risk ratios calculated from event counts were used when hazard ratios were unavailable. Effects were log-transformed, such that negative values favored fewer clinical events with the intervention. HRs and risk ratios (RRs) were combined as a pragmatic approximation on the log-relative-effect scale and examined separately in sensitivity analyses. No otherwise eligible primary weight comparison was excluded solely because both groups had zero clinical events. In 3 T, sparse 30-day arm-level events and incomplete outcome status information did not permit recovery of a pairwise clinical effect and uncertainty; exclusion was not based simply on refusal to apply a continuity correction. DOSE and ROSE-AHF contributed more than one eligible comparison with shared participants or controls; dependence was handled by trial-clustered resampling and examined by retaining one comparison per trial. TACTICS-HF reported Kaplan–Meier (KM) risks, not event counts. Its 30-day risk ratio was calculated as 0.33/0.29, with an approximate log-SE from the independent arm log-risk intervals (0.25–0.42 and 0.22–0.38); it is labeled KM-RR and excluded in a separate sensitivity analysis (13). SECRET of CHF event percentages corresponded to the treated populations (119/125); this denominator interpretation was retained explicitly (14).

### Risk of bias

Risk of bias was evaluated for quantitatively analyzed outcomes using the Cochrane RoB 2 domains: randomization, deviations from intended interventions, missing outcome data, outcome measurement, and selection of the reported result (15). Open-label treatment was not automatically judged high risk; concern was increased when lack of masking could plausibly affect cointerventions, follow-up, or non-blinded outcome ascertainment. Retrospective registration, premature termination, and incomplete reporting informed domain judgments.

### Statistical analysis

For comparison j, the observed weight effect x_obs,j was modeled as x_obs,j|xj ∼ N(xj, sxj^2^), and the observed log clinical effect y_obs,j as y_obs,j|xj ∼ N(*α* + *β*xj, syj^2^ + *τ*^2^). The latent true weight effect xj was profiled conditionally; *α*, *β*, and log(*τ*) were estimated by maximum likelihood. Weighting arose from sxj^2^ and syj^2^ + *τ*^2^ in the joint likelihood; no additional sample-size or inverse-variance weight was imposed. *β* is the ecological change in log clinical effect per additional 1 kg of between-group weight loss, and exp(*β*) is the relative clinical effect ratio per kilogram. A ratio below 1 means that comparisons producing more weight loss tended to show more favorable relative clinical effects; it does not estimate an individual patient's risk reduction from losing 1 kg. Source trials did not report the within-comparison covariance between weight and clinical effect estimates, so covariance was assumed to be zero.

This model was chosen to estimate an exploratory treatment-effect association while accounting for sampling error in both extracted effects. Formal meta-analytic surrogate validation commonly quantifies trial-level predictive association, including trial-level R^2^, within hierarchical or joint models (16, 17). With only 10 independent trials, heterogeneous clinical endpoints and follow-up, mixed treatment mechanisms, and no individual participant data, such metrics would be unstable and could imply a level of validation unsupported by the evidence. We therefore did not estimate trial-level R^2^ or individual-level surrogacy and did not interpret the errors-in-variables (EIV) slope as mediation or formal surrogate validation.

Uncertainty was estimated with 10,000 successful non-parametric bootstrap fits clustered by trial ID, preventing multiple comparisons from one trial from being treated as fully independent. Because the primary analysis contained only 10 independent trial clusters, we inspected the empirical slope distribution and performed a leave-one-trial-out analysis. The percentile bootstrap was treated as an exploratory small-sample uncertainty analysis rather than a definitive calibration procedure. Spearman’s correlation was reported descriptively. We added a trial-level jackknife standard error with a t-based interval. Additional *post hoc* exploratory restrictions considered HF event endpoints, 60–90-day follow-up, HRs only, RRs only, exclusion of high-risk trials, and low-risk trials only. A profile likelihood analysis was performed to assess residual *τ*; the approximate likelihood support upper bound used the *χ*^2^1 95% cutoff (3.84). Prespecified sensitivity analyses retained one comparison per trial, pharmacological trials only, and markers measured at 48–96 h. Additional exploratory analyses excluded comparisons reporting mortality alone and included weight measured at 120 h or day 15. Funnel plots and small-study publication bias tests were not performed because the objective was association rather than pooled treatment efficacy, and the number of comparisons was small. Analyses were conducted in 64-bit Python 3.12.14 with NumPy 2.5.1, SciPy 1.18.0, and pandas 3.0.3. Effect estimates and intervals guided interpretation; *P*-values were descriptive without adjustment for multiplicity. Further *post hoc* checks excluded the KM risk approximation; excluded DOSE, SECRET of CHF, TACTICS-HF, and CLOROTIC because of denominator or interval approximations; and increased the DOSE/SECRET weight SEs by 25%. Numerically extreme bootstrap slopes were retained and their frequency reported; optimizer convergence alone did not establish reliable identification.

## Results

### Study selection and characteristics

The search identified 9,326 records: 3,044 from PubMed, 5,490 from OpenAlex, and 792 from ClinicalTrials.gov. After deduplication, 7,300 records underwent title/abstract screening, 252 entered the candidate set, and 32 full-text reports representing 29 distinct trials were assessed. Five trials were excluded after full-text analysis: RAPID-CHF, ULTRADISCO, FASTR, and CAVA-ADHF did not report an eligible clinical outcome at ≥30 days; a 2026 randomized trial of albumin plus furosemide reported neither an eligible objective decongestion measure nor an eligible follow-up clinical outcome. The qualitative synthesis included 24 trials reported in 27 articles. Twelve comparisons from 10 trials entered the primary-weight meta-regression; adding the out-of-window Palazzuoli (2014) and EMPULSE weight measures yielded 14 comparisons from 12 trials in the expanded analysis (Figure 1).

**Figure 1:**
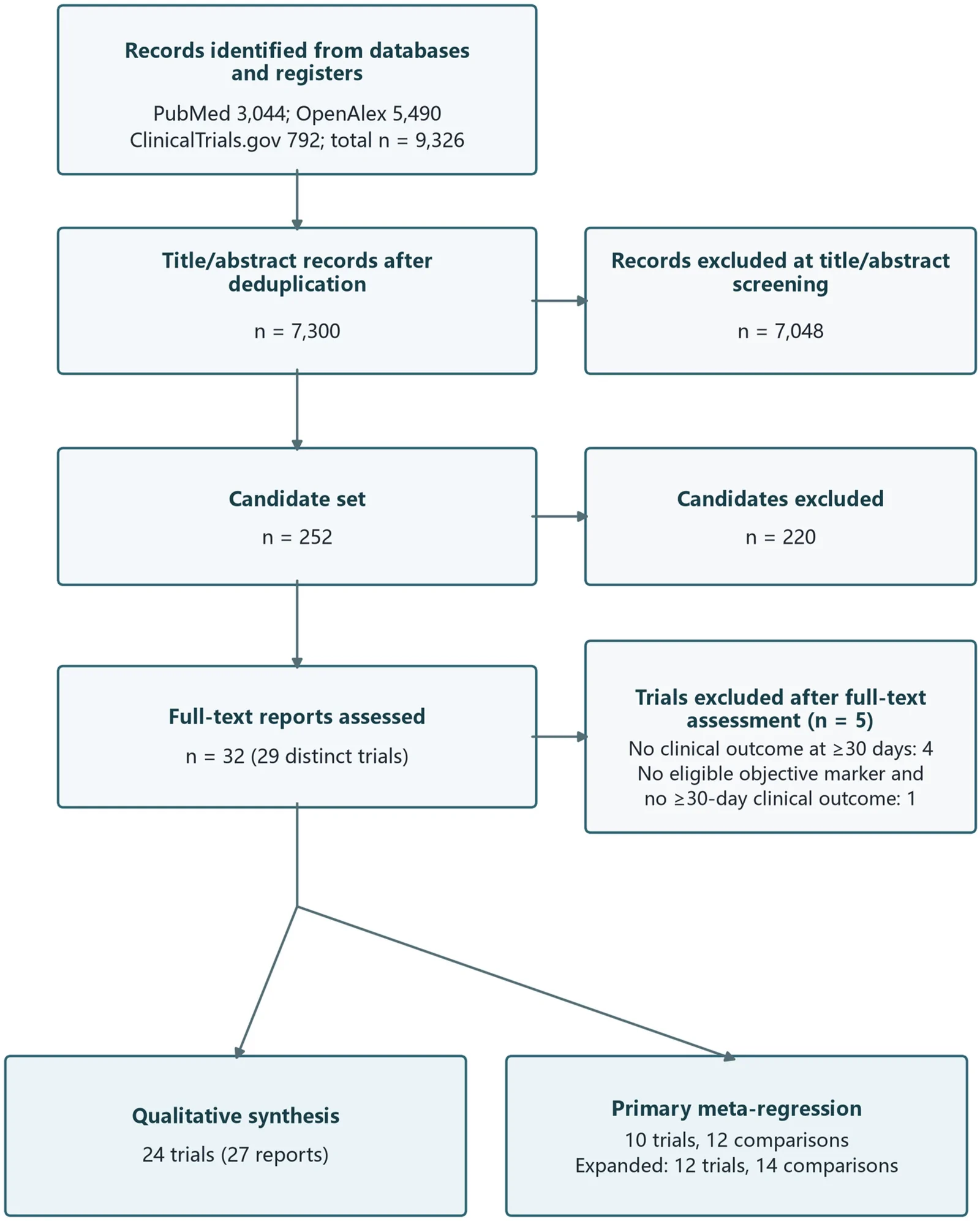
PRISMA flow of study selection.

Trials were published from 2007 through 2025 and ranged from 59 to 4,133 randomized participants. Interventions included loop diuretic dose and delivery strategies, thiazide or mineralocorticoid receptor antagonist combinations, vasopressin antagonists, sodium-glucose cotransporter 2 inhibitors, acetazolamide, low-dose dopamine or nesiritide, ultrafiltration, and urinary-sodium- or ultrasound-guided treatment. Body weight was the most consistently reported marker; urine output, urinary sodium, natriuretic peptides, and imaging-defined residual congestion varied substantially in definition and timing (Table 2). Table 3 describes age, sex, ejection fraction, renal function, and HF history in the 12 quantitatively analyzed trials, retaining source-specific summaries and identifying unavailable fields. These are descriptive trial characteristics, not pooled patient-level estimates.

**Table 2:** Principal characteristics of included trials.

Trial (year)	N	Randomized comparison	Short-term marker	Clinical follow-up outcome
UNLOAD (2007) (6)	200	Ultrafiltration vs. IV diuretics	48-h weight/fluid loss	90-day HF rehospitalization/death
EVEREST (2007) (4, 5)	4,133	Tolvaptan vs. placebo	Day-1/7 weight, edema	Median 9.9-month death/CV death or HFH
DOSE (2011) (2, 3)	308	High vs. low dose; infusion vs. bolus	72-h weight/fluid loss	60-day death/hospitalization/ED visit
CARRESS-HF (2012) (7)	188	Ultrafiltration vs. stepped drugs	72/96-h weight	60-day death/HF hospitalization
ROSE-AHF (2013) (18)	360	Dopamine or nesiritide vs. placebo	72-h weight/urine	60-day death
Palazzuoli (2014) (19)	82	Continuous vs. bolus furosemide	120-h weight/urine	180-day death/rehospitalization
DAD-HF II (2014) (20)	161	High/low furosemide ± dopamine	Urine output/weight	60-day death/readmission
AVOID-HF (2016) (21)	224	Adjustable UF vs. adjustable IV diuretics	Weight/fluid removal	90-day HF events/death
TACTICS-HF (2017) (22)	257	Tolvaptan vs. placebo	48/72-h weight/fluid	30-day death/hospitalization
SECRET of CHF (2017) (14)	250	Tolvaptan vs. placebo	24-h weight	30-day death/HF hospitalization
ATHENA-HF (2017) (23)	360	High-dose spironolactone vs. usual care	96-h weight/NT-proBNP	30-day HF visit/hospitalization/death
3T (2020) (24)	60	Metolazone vs. chlorothiazide vs. tolvaptan	48-h weight/urine	30-day events (exploratory)
EMPA-RESPONSE-AHF (2020) (25)	80	Empagliflozin vs. placebo	4-day urine/weight	60-day death/HF event/readmission
Early UF (2020) (26)	100	Early UF vs. torasemide + tolvaptan	Day-4/8 weight	1- and 3-month readmission/death
EMPAG-HF (2022) (27)	59	Empagliflozin vs. placebo	5-day urine output	30-day mortality
EMPULSE (2022) (28, 29)	530	Empagliflozin vs. placebo	Day-15 weight/congestion	90-day CV death/HF event
ADVOR (2022) (30)	519	Acetazolamide vs. placebo	Successful decongestion/urine/Na	3-month death/HF hospitalization
CLOROTIC (2023) (31)	230	Hydrochlorothiazide vs. placebo	72-h weight/urine	90-day death/rehospitalization
PUSH-AHF (2023) (32)	310	Natriuresis-guided vs. usual care	24-h natriuresis; 72-h weight	180-day death/HF hospitalization
DAPA-RESPONSE-AHF (2023) (33)	87	Dapagliflozin vs. placebo	4-day urine/NT-proBNP	30-day death/readmission
DICTATE-AHF (2024) (34)	240	Dapagliflozin vs. usual care	Diuretic efficiency/urine Na	30-day readmission/death
CAVAL US-AHF (2024) (35)	60	IVC + lung US guidance vs. usual care	Residual US congestion	90-day HF visit/readmission/death
Urinary sodium pilot (2025) (36)	60	UNa-guided vs. clinical titration	48-h weight/congestion	30-day death/readmission
AVANTI (2025) (37)	483/286^a^	Pecavaptan adjunct/replacement strategies	30-day weight	30-day congestion-related events/HFH

CV, cardiovascular; ED, emergency department; HFH, heart failure hospitalization; IV, intravenous; IVC, inferior vena cava; UF, ultrafiltration; UNa, urinary sodium; US, ultrasound.

EMPULSE weight at day 15 entered only the expanded analysis.

a AVANTI randomized 483 participants in Part A; 286 eligible participants were subsequently rerandomized in Part B. These counts must not be added.

**Table 3:** Baseline characteristics of the quantitatively analyzed trials.

Trial and arm order	Age (years)	Sex	LVEF (%)	Renal function	HF history/presentation
UNLOAD (6) UF/diuretics 100/100	62 ± 15/63 ± 14	Men 70%/68%	71% had LVEF≤40% (overall)	Cr 1.5 ± 0.5/1.5 ± 0.5 mg/dL	*De novo* proportion not recovered
EVEREST (5) Tolvaptan/placebo 2072/2061	65.9 ± 11.7/65.6 ± 12.0	Men 73.4%/75.4%	27.5 ± 8.0/27.5 ± 8.2	Chronic renal insufficiency 26.5%/27.1%	Previous HF hospitalization 79.2%/78.1%
DOSE (2) 308 unique participants; factorial arms^a^	Arm means 65.8–66.2	Men 73%–74%	Arm means 33–36	Cr 1.5 ± 0.5 mg/dL in each arm	HF hospitalization in prior year 71%–77%
CARRESS-HF (7) UF/drug strategy 94/94	69 (61–78)/66 (57–78)	Men 78%/72%	30 (20–52)/35 (25–55)	Cr 1.90 (1.57–2.37)/2.09 (1.71–2.65) mg/dL	HF hospitalization in prior year 75%/79%
ROSE-AHF (18) Dopamine/pooled placebo/nesiritide 122/119/119	71 (63–80)/70 (62–78)/69 (59–79)	Men 69%/75%/76%	35 (23–52)/30 (20–50)/35 (20–55)	eGFR 45.5 (31.2–59.2)/44.5 (32.6–53.0)/43.8 (34.7–55.3)	HF hospitalization in prior year 65%/67%/69%
Palazzuoli (2014) (19) Continuous/bolus 43/39	80 ± 4/79 ± 5^b^	Women 24/18 (counts)	34.3 ± 10/35.8 ± 8	eGFR 43.2 ± 7.6/45.7 ± 8.7	*De novo* proportion not recovered
TACTICS-HF (13, 22) Tolvaptan/placebo 129/128	66.42 ± 12.9/62.97 ± 15.7	Women 44/42 (counts)	All EF eligible; baseline summary not recovered	Cr > 3.5 mg/dL excluded (eligibility; not a baseline value)	Required prior daily oral loop diuretic
SECRET of CHF (14) Tolvaptan/placebo 122/128 randomized	70 ± 11/67 ± 13	Women 30%/35%	35 ± 16/33 ± 17	eGFR 48 ± 19/47 ± 19	Required HF treatment for≥1 month
ATHENA-HF (23) High-dose/usual care 182/178	65 (57–76)/65 (54–74)	Women 36%/36%	35 (21–50)/30 (20–45)	eGFR 58 (45–75)/55 (46–71)	HF hospitalization in prior year 66%/64%
Early UF 2020 (26) UF/torasemide + tolvaptan 40/60	70.60 ± 10.44/73.52 ± 9.83	Men 55%/55%	Not measured (reported)	Cr 155.70 ± 72.05/128.30 ± 82.66 µmol/L	Prior HF 57.5%/63.3%
EMPULSE (28) Empagliflozin/placebo 265/265	71 (62–78)/70 (59–78)	Women 32.5%/35.1%	31 (23–45)/32 (22.5–49)	eGFR 50 (36–65)/54 (39–70)	*De novo* HF 33.2%/32.8%
CLOROTIC (31) HCTZ/placebo 114/116	83 (78–87)/82 (75–87.5)	Women 39.5%/56.9%	55 (40–63)/57 (40–63)	eGFR 43.0 (32.0–58.2)/43.5 (34.8–58.0)	Chronic HF; prior loop diuretic treatment required

EF, ejection fraction; LEVF, left ventricular ejection fraction. Values are mean ± SD or median (IQR), as reported; no pooled baseline values were imputed. eGFR is in mL/min/1.73 m^2^. Sex is retained as men or women to avoid unreported conversions. “Not recovered” denotes an unverified field, not a claim that the source did not report it. HF hospitalization history is not equivalent to the *de novo*/chronic HF classification.

a DOSE values are ranges across its four marginal factorial groups, not pooled estimates or 616 independent participants.

b Table 2 of the original Palazzuoli et al. (2014) report gives 80 ± 4/79 ± 5 years, whereas the text gives an overall 72 ± 8 years; the source discrepancy is retained explicitly. This table covers the 12 quantitatively analyzed trials; Table 2 describes all 24 included trials.

### Risk of bias

Of the 12 trials contributing to the primary or expanded quantitative analysis, 8 were judged at low overall risk of bias, UNLOAD and CARRESS-HF had some concerns, and Early UF 2020 and Palazzuoli (2014) were at high risk. High-risk judgments reflected potential deviations in open-label studies, retrospective registration or selective reporting, and incomplete reporting of secondary clinical outcomes in small trials (Figure 2).

**Figure 2:**
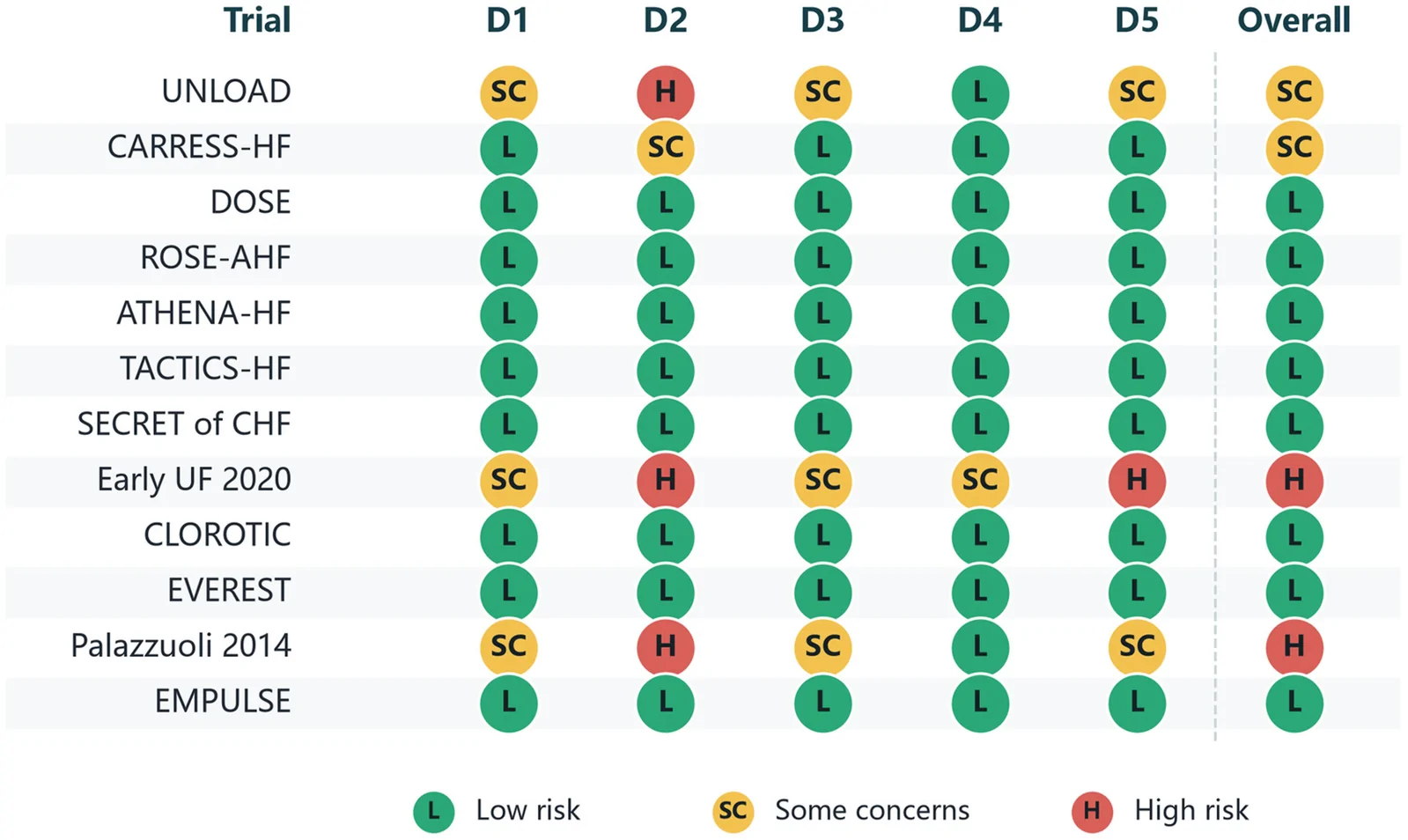
Outcome-level risk of bias for quantitatively analyzed trials. D1 randomization; D2 deviations; D3 missing data; D4 measurement; D5 selective reporting.

### Primary analysis of weight and clinical effects

The 12 primary comparisons from only 10 independent trials paired weight measured at 24–96 h with clinical follow-up ranging from 30 days to a median of 9.9 months (approximately 302 days). Between-group weight effects ranged from 0.263 kg less weight loss to 2.30 kg greater weight loss with the intervention, while corresponding clinical effects ranged from clearly favorable to unfavorable (Table 4). Five comparisons used HRs, six used count-based RRs, and one used an approximate KM-RR. Table 5 lists the selected endpoint and selection rationale for each quantitative trial.

**Table 4:** Paired body-weight and clinical treatment effects.

Trial	Comparison	Time	Extra weight loss, kg ± SE	Clinical endpoint	Follow-up	Clinical effect (95% CI)	Set
UNLOAD	Ultrafiltration vs. intravenous diuretics	48 h	1.90 ± 0.51	HF rehospitalization	90 days	RR 0.56 (0.33–0.96)	Primary
CARRESS-HF	Ultrafiltration vs. stepped pharmacologic therapy	72 h	0.41 ± 0.63	HF hospitalization	60 days	RR 0.99 (0.60–1.62)	Primary
DOSE	High-dose loop diuretic vs. low-dose loop diuretic	72 h	1.18 ± 0.47	Death hospitalization or emergency visit	60 days	HR 0.83 (0.60–1.16)	Primary
DOSE	Continuous infusion vs. bolus every 12 h	72 h	0.59 ± 0.47	Death hospitalization or emergency visit	60 days	HR 1.15 (0.83–1.60)	Primary
ROSE-AHF	Low-dose dopamine vs. placebo	72 h	−0.15 ± 0.44	All-cause death	60 days	RR 0.89 (0.41–1.95)	Primary
ROSE-AHF	Low-dose nesiritide vs. placebo	72 h	−0.26 ± 0.44	All-cause death	60 days	RR 0.67 (0.28–1.57)	Primary
ATHENA-HF	High-dose spironolactone vs. usual care	96 h	0.27 ± 0.48	HF hospitalization emergency visit or death	30 days	HR 1.22 (0.68–2.19)	Primary
Palazzuoli (2014)	Continuous infusion vs. bolus intermittent infusion	120 h	0.60 ± 0.48	Death or rehospitalization	180 days	HR 2.91 (1.28–6.63)	Expanded only
TACTICS-HF	Tolvaptan vs. placebo	72 h	1.21 ± 0.48	Death/rehospitalization, including unscheduled clinic/ED visits	30 days	KM-RR 1.14 (0.78–1.66)	Primary
SECRET of CHF	Tolvaptan vs. placebo	24 h	1.42 ± 0.25	HF rehospitalization or death	30 days	RR 0.92 (0.47–1.80)	Primary
Early UF 2020	Early ultrafiltration vs. torasemide plus tolvaptan	72 h	2.30 ± 0.61	Readmission	90 days	RR 0.55 (0.27–1.10)	Primary
CLOROTIC	Hydrochlorothiazide vs. placebo	72 h	1.14 ± 0.36	All-cause rehospitalization	90 days	HR 1.25 (0.81–1.93)	Primary
EVEREST	Tolvaptan vs. placebo	24 h	0.79 ± 0.06	CV death or HF hospitalization	9.9 months	HR 1.04 (0.95–1.14)	Primary
EMPULSE	Empagliflozin vs. placebo	Day 15	1.97 ± 0.45	CV death or HF event	90 days	HR 0.69 (0.45–1.08)	Expanded only

SE, standard error; HR, hazard ratio; RR, risk ratio. Positive weight values indicate greater loss with the intervention; HR or RR <1 favors fewer clinical events. Some RRs and confidence intervals were calculated from event counts. KM-RR denotes an approximate fixed-time Kaplan–Meier risk ratio, not a count-based RR or HR. Weight SE approximations in DOSE, SECRET of CHF, and CLOROTIC are described in the Methods section and tested in Table 1. Early UF day 4 corresponds to approximately 72 elapsed hours after three treatment days; exact clock time was not reported.

**Table 5:** Audit of clinical endpoint selection for quantitative comparisons.

Trial	Eligible reported clinical outcome(s)/follow-up	Selected quantitative endpoint	Selection rationale
UNLOAD	HF rehospitalization and death/90 days	HF rehospitalization	Recoverable HF event component with more events
CARRESS-HF	Death and HF hospitalization/60 days	HF hospitalization	Recoverable HF event component
DOSE	Death, hospitalization, or ED visit/60 days	Reported composite HR	Highest-ranked reported composite
ROSE-AHF	All-cause death/60 days in paired report	All-cause death	Only recoverable eligible endpoint for paired comparison
ATHENA-HF	HFH, urgent HF visit, or death/30 days	Reported composite HR	Highest-ranked reported composite
TACTICS-HF	Death/rehospitalization, including unscheduled clinic/ED visits/30 days	Same composite; approximate KM-RR	Public registry arm KM risk intervals; not event counts
SECRET of CHF	death or HFH/30 days	Reported composite RR	Highest-ranked HF event composite
Early UF 2020	Readmission and death/90 days	Readmission	Recoverable event component with adequate counts
CLOROTIC	Death and all-cause rehospitalization/90 days	All-cause rehospitalization HR	Reported time-to-event estimate
EVEREST	All-cause death; CV death or HFH/median 9.9 months	CV death or HFH HR	Highest-ranked HF event composite
Palazzuoli (2014)	Death or rehospitalization/180 days	Composite HR	Univariate treatment HR; avoids post-treatment adjustment; expanded set
EMPULSE	CV death or HF event/90 days	Composite HR	Expanded timing analysis only

The errors-in-variables meta-regression slope was −0.302. Each additional 1-kg between-group difference in short-term weight loss corresponded to a relative clinical effect ratio of 0.74 (10,000-fit percentile 95% interval, 0.56–1.26; trial-jackknife 95% interval, 0.49–1.11). The estimated residual between-comparison SD was *τ* = 0.000016 on the log clinical effect scale; its boundary-near value should not be interpreted as proof of homogeneity in this small model. Although the point estimate was directionally consistent with fewer events after greater weight loss, the interval allowed clinically meaningful benefit, no association, or an adverse relation. Spearman’s *ρ* was −0.308. The bootstrap median clinical effect ratio was 0.74. UNLOAD and the small Early UF trial occupied the region of both greater weight loss and fewer events, whereas EVEREST, CLOROTIC, TACTICS-HF, and some DOSE comparisons showed improved weight but neutral or directionally unfavorable clinical effects (Figure 3).

**Figure 3:**
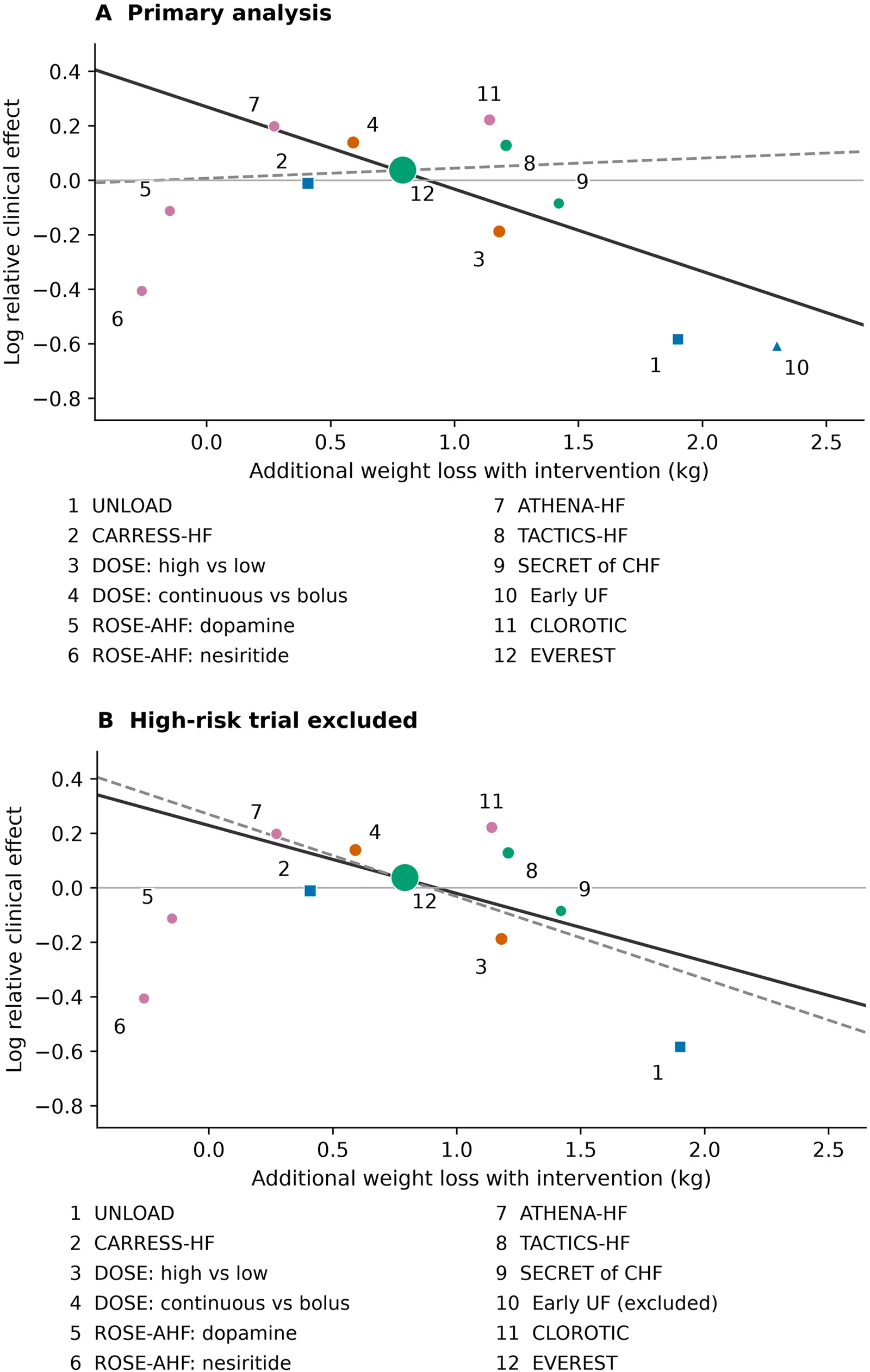
Exploratory trial-level association. **(A)** Primary model (solid line) and pharmacological-only fit (dashed). **(B)** High-risk trial-excluded model (solid) with the primary fit for reference (dashed). Point numbers use the trial key below each panel. Blue indicates ultrafiltration, orange loop diuretic strategies, green vasopressin antagonists, and purple other drugs. Circles, squares, and triangles indicate low risk, some concerns, and high risk of bias, respectively. Size reflects clinical effect precision. Positive x values indicate greater weight loss; negative y values indicate fewer clinical events.

### Sensitivity analyses

The original 48–96-h window gave a ratio of 0.74 (0.48–1.36). Estimates were 0.69 with one comparison per trial and 1.04 in pharmacological trials. The HF event subset gave 0.64 (bootstrap interval, 0.57–0.99; jackknife, 0.34–1.21), illustrating sensitivity to the uncertainty method in only five trials. The 60–90-day estimate was 0.75; count-based RRs gave 0.82. The HR-only estimate was 0.68, but its four-cluster bootstrap was numerically unstable (34/1,000 slopes had absolute values >20); its percentile interval is not interpreted as reliable precision. Excluding Early UF gave 0.78 (Figure 3B). The low-risk-only and pharmacological-only sets were identical, so mechanism and risk of bias could not be separated (Table 1).

Exclusion of the ROSE-AHF comparisons that reported all-cause mortality alone produced an exploratory ratio of 0.64 (percentile 95% interval, 0.51–1.03), but materially changed the clinical endpoint composition and removed neutral comparisons with imprecise mortality estimates. The expanded analysis incorporating Palazzuoli (2014) at 120 h and EMPULSE at day 15 yielded 0.70 (percentile 95% interval, 0.37–0.96). Neither exploratory result constituted robust prespecified evidence: The first was sensitive to outcome selection, while the second mixed different marker time scales and included therapy with disease-modifying effects. These analyses cannot validate weight as a surrogate and are not used to support the principal conclusion (Table 1). Excluding the KM risk approximation yielded 0.73 (0.59–1.27); excluding all designated denominator/interval approximations yielded 0.74 (0.59–1.30). Inflating the uncertain weight SEs and retaining the previous CARRESS/TACTICS time points gave 0.74 and 0.75, respectively.

The analysis retained 10,000 successful primary trial cluster bootstrap fits. The empirical distribution was discrete and irregular, as expected with 10 trial clusters. Leave-one-trial-out clinical effect ratios ranged from 0.64 to 0.82 per kg, preserving the favorable direction but showing material influence on magnitude; the largest individual shifts followed omission of ROSE-AHF and UNLOAD. The profile likelihood placed *τ* at the boundary; the approximate 95% profile upper limit was 0.130, indicating that clinically relevant residual heterogeneity cannot be excluded (Figure 4). One primary bootstrap fit returned an extreme slope (−601.98); it was retained in the percentile calculation. The bootstrap and jackknife intervals both crossed null in the primary analysis.

**Figure 4:**
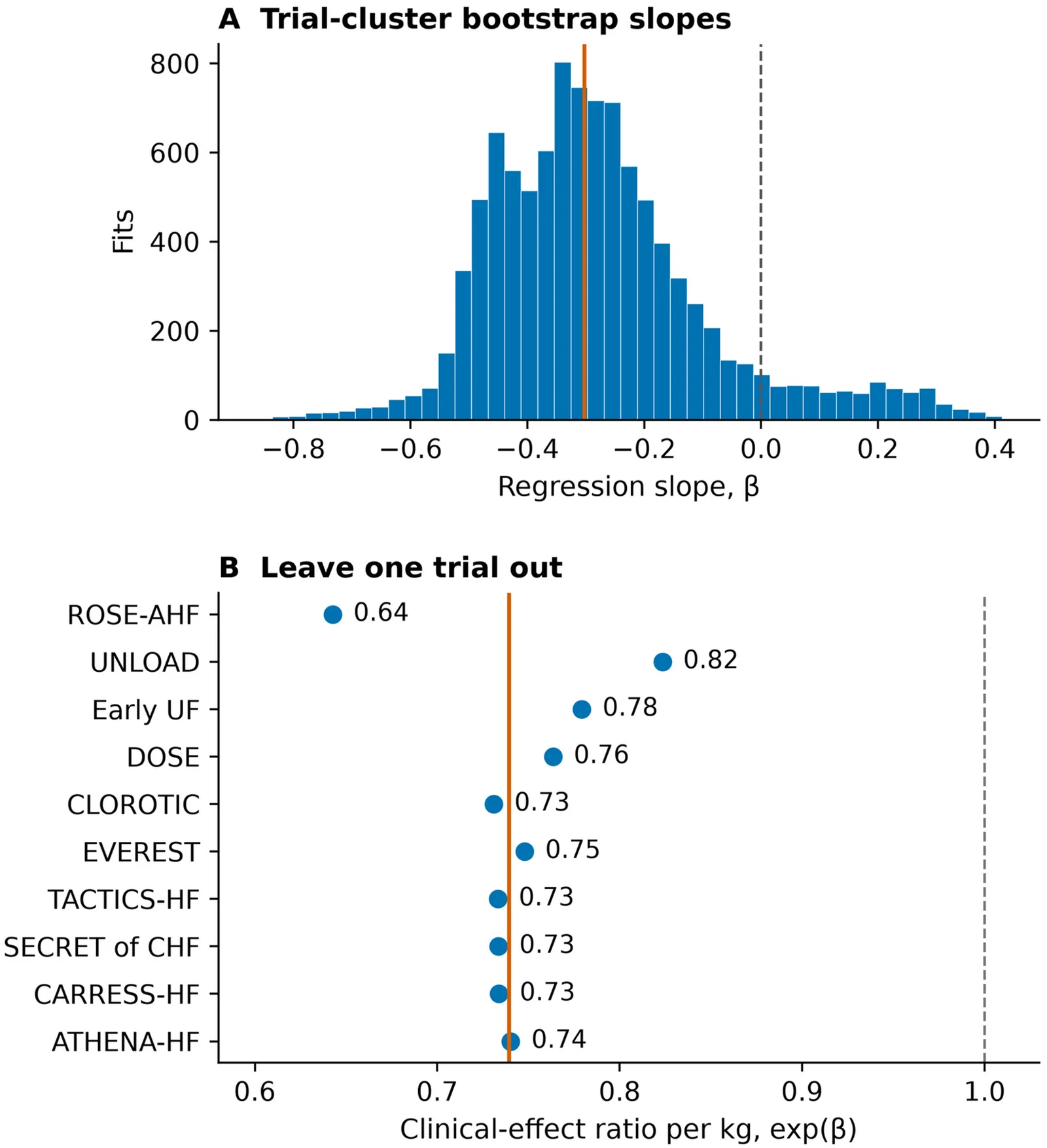
Small-cluster uncertainty and influence diagnostics. **(A)** shows the 10,000-successful-fit trial cluster bootstrap distribution; **(B)** shows the clinical effect ratio after omission of each trial. **(A)** displays the central 99.8% of slopes for legibility (20 tail fits outside the plotting range); all 10,000 fits, including the extreme slope, were retained for inference. The orange line marks the primary estimate.

### Concordance of other decongestion measures with clinical outcomes

Non-weight markers showed similar mechanism and study size dependence. In ADVOR, acetazolamide increased successful decongestion, urine volume, and natriuresis, but did not reduce death or heart failure hospitalization at 3 months (30). PUSH-AHF increased 24-h natriuresis by 63 mmol with natriuresis-guided treatment, yet did not reduce death or heart failure hospitalization through 180 days (HR, 0.92; 95% CI, 0.62–1.38) (32). These trials demonstrate that improved early natriuretic efficiency can benefit the inpatient decongestion process without being sufficient for post-discharge clinical benefit.

Signals of concordance were present in small studies. EMPA-RESPONSE-AHF increased urine output and reported fewer 60-day composite events, but included only 80 participants (25). CAVAL US-AHF markedly reduced ultrasound-defined residual congestion at discharge and reported a 90-day composite in 4 of 30 vs. 11 of 30 participants (35). Conversely, EMPAG-HF increased 5-day urine output but accrued too few 30-day deaths to establish a clinical relation (27). DICTATE-AHF did not consistently improve diuretic efficiency or natriuresis and did not show a clear 30-day clinical advantage (34). A 60-patient urinary-sodium-guided pilot improved 48-hour weight loss, congestion score, and kidney safety, but not 30-day death or readmission (36) (Table 6).

**Table 6:** Concordance of non-weight decongestion and clinical effects.

Trial	Decongestion marker	Marker effect	Clinical endpoint	Clinical effect	Concordance
ADVOR (30)	Urine output/natriuresis; successful decongestion	Greater	Death or HF hospitalization	No clear benefit demonstrated	Discordant
PUSH-AHF (32)	24-h natriuresis	Greater	180-day death or HF hospitalization	No clear benefit demonstrated	Discordant
EMPA-RESPONSE-AHF (25)	Urine output	Greater	60-day death/HF worsening/readmission	Fewer events	Directionally concordant; underpowered
EMPAG-HF (27)	5-day urine output	Greater	30-day mortality	Uncertain	Indeterminate/underpowered
DICTATE-AHF (34)	Diuretic efficiency/natriuresis	No consistent improvement	30-day readmission/death	No clear benefit demonstrated	Concordant null
CAVAL US-AHF (35)	Residual ultrasound congestion	Lower	90-day HF visit/readmission/death	Fewer events	Directionally concordant; underpowered
Urinary sodium-guided pilot (36)	48-h weight/congestion score	Greater	30-day death/readmission	No clear benefit demonstrated	Discordant; underpowered

This is a structured qualitative synthesis; event differences in small pilot trials do not establish surrogacy. The categories are descriptive and do not have the evidential status of the quantitative weight analysis. “Discordant” denotes marker improvement without demonstrated clinical benefit, not necessarily an unfavorable clinical point estimate; “concordant null” denotes no clear improvement in either domain, not equivalence; “directionally concordant; underpowered” denotes favorable marker and clinical point estimates with limited event evidence; and “indeterminate/underpowered” denotes insufficient clinical event information for classification.

## Discussion

This paired trial-level synthesis produced three principal findings. First, greater weight loss at 24–96 h was directionally associated with fewer subsequent clinical events, but the primary association across only 10 independent trials was statistically uncertain. Second, the relation was nearly absent when analysis was restricted to pharmacological trials and was influenced by a small number of ultrafiltration studies. Third, qualitative evidence for urinary sodium, urine output, natriuretic peptides, and ultrasound markers was also discordant: better early decongestion could coexist with later benefit, but was not sufficient for it. These measures remain useful indicators of treatment response and congestion status, but the randomized trial-level evidence evaluated here does not establish any single one as a validated clinical surrogate.

The result highlights the distinction between patient-level prognostic value and trial-level surrogacy. Residual congestion is associated with death and readmission because it reflects severity and incomplete recovery. That observation does not imply that every treatment-induced reduction in a congestion marker will proportionally improve outcomes. Formal surrogate validation requires the treatment effect on the candidate surrogate to reliably predict the treatment effect on the true endpoint, ideally across many trials and mechanisms and with both individual- and trial-level evidence (16). Our aggregate data analysis evaluates only the trial-level relation and cannot complete such validation.

Body weight is particularly vulnerable to mechanistic ambiguity. Tolvaptan predominantly promotes water excretion; EVEREST showed early weight and symptom improvement without long-term clinical benefit (4, 5). Ultrafiltration removes isotonic fluid directly, but its net effect depends on removal rate, vascular refilling, renal perfusion, catheter complications, and selection of the target population. The contrast between UNLOAD and CARRESS-HF illustrates that conditionality (6, 7). Sodium-glucose cotransporter 2 inhibitors have modest diuretic effects along with renal and metabolic actions. EMPULSE showed concordant day-15 weight and 90-day clinical effects (28, 29), but the clinical benefit cannot be assumed to be mediated by weight loss. Collapsing these mechanisms into an effect “per kilogram” sacrifices causal interpretability.

The divergence between the overall and pharmacological-only estimates is clinically important. The primary point estimate was 0.74 per additional kilogram, compared with 1.04 among drug trials. The open-label, retrospectively registered Early UF 2020 study was small and contributed a large favorable event contrast (26). However, the largest leave-one-trial-out shifts followed omission of ROSE-AHF and UNLOAD; Early UF produced a smaller shift. The low-risk-only set was identical to the pharmacological-only set (Table 1). With only 10 trials in the primary analysis, intervention class and study quality remain confounded even after errors-in-variables modeling and trial-clustered bootstrapping. A regression association cannot therefore be interpreted as evidence that weight change itself mediates clinical benefit.

Urinary sodium may be closer than weight to the immediate pharmacodynamic action of loop diuretics, yet it also failed to demonstrate sufficient surrogacy. PUSH-AHF established that a natriuresis-guided algorithm can increase sodium excretion without reducing the 180-day death or heart-failure-hospitalization composite (32). A recent meta-analysis similarly found improved urine output and natriuresis with guided strategies but limited clinical event evidence (9). Urinary sodium is well suited as an operational marker for detecting insufficient response and prompting treatment escalation. Its value may lie in reducing delay, standardizing dose intensification, and improving inpatient efficiency rather than guaranteeing lower mortality.

Imaging-defined residual congestion offers complementary information. Lung B-lines and inferior vena cava indices can identify pulmonary interstitial fluid or venous pressure not captured by weight. CAVAL US-AHF reported both less residual congestion and fewer 90-day events, but was a 60-patient pilot (35). As contextual evidence outside the formal synthesis, the larger multicenter CAVA-ADHF trial did not improve NT-proBNP or other inpatient measures (38) and was excluded because it did not report an eligible ≥30-day clinical endpoint. Ultrasound should therefore be considered one component of multidimensional assessment rather than a stand-alone treatment target.

### Clinical implications

The absence of a validated single surrogate does not make decongestion markers clinically unhelpful. At the bedside, serial weight on the same scale should be interpreted with the help of symptoms, examination, early urine output and urinary sodium after loop diuretic dosing, renal electrolyte and hemodynamic safety, and, when available, discharge ultrasound evidence of residual congestion (1, 32, 35). An inadequate early natriuretic or diuretic response can trigger treatment reassessment, but no isolated weight, sodium, or ultrasound value should determine discharge. Discharge readiness should instead reflect integrated improvement in congestion and perfusion, a safe oral regimen, and a post-discharge monitoring plan.

Future AHF trials should move beyond choosing between weight and urine output as a single primary marker. A core set should include weight at fixed times, 6- or 24-h urine output, early spot urinary sodium and 24-h total sodium excretion, a standardized congestion score, kidney and electrolyte safety, discharge residual congestion, and at least 90-day mortality and heart failure events. Surrogate validation would require harmonized clinical endpoints and follow-up, individual participant data meta-analysis across sufficiently numerous and mechanistically diverse randomized trials, and out-of-sample prediction of clinical treatment effects. Inpatient process endpoints and post-discharge clinical endpoints should remain distinct rather than being collapsed into a single label of “successful decongestion.”

The novelty of this study lies in its question and unit of analysis. Previous reviews pooled the average effect of treatments on weight or urine output, or the average effect of a guidance strategy on events. We instead paired both effects within the same randomized comparison and asked whether the magnitude of treatment-induced decongestion tracked the magnitude of treatment-induced clinical benefit. This structure more closely reflects the trial-level concept of a surrogate and exposes structural deficiencies in the evidence base: missing clinical outcomes, inconsistent marker timing, and heterogeneity of treatment mechanisms.

Several limitations should temper interpretation. First, direct database searching was limited to PubMed, OpenAlex, and ClinicalTrials.gov; Embase and CENTRAL were unavailable, and trials may have been missed despite citation chasing. Second, screening, extraction, and risk-of-bias assessment were performed by one investigator with a same-investigator verification pass, not independently duplicated. Third, the primary model contained only 12 comparisons from 10 trials, limiting power and precluding reliable assessment of non-linearity, treatment–class interaction, or publication bias. The trial cluster bootstrap consequently sampled a discrete, occasionally ill-conditioned small-cluster distribution, so its confidence interval and *P*-value require caution despite the added distributional and leave-one-out diagnostics. The near-boundary *τ* estimate was also weakly identified. Fourth, some weight SEs used approximate denominators or interval conversions, and one clinical effect used approximate KM risk intervals; clinical effects mixed hazard and risk ratios, and follow-up times and composite definitions differed. Within-comparison covariance between marker and clinical effect estimates was unavailable and assumed to be zero; weight measurement conditions were incompletely standardized. Fifth, open-label ultrafiltration trials combined high leverage with higher risk of bias. Sixth, aggregate trial data cannot separate patient-level prognostic effects, mediation, and ecological confounding. Seventh, the review was not prospectively registered in PROSPERO; locally saved protocol files and hashes improve auditability but do not replace public prospective registration. Selective availability of paired marker and clinical effects may further bias the analyzable evidence set, even when a trial is published. Finally, statistically positive exploratory analyses were sensitive to endpoint and time window choices and should be viewed as hypothesis-generating.

## Conclusions

Across only 10 independent randomized AHF trials, short-term weight loss showed only a directional, mechanism-dependent, and non-robust trial-level relation with subsequent clinical benefit. The randomized trial-level evidence evaluated here is insufficient to validate short-term weight change or any single evaluated decongestion marker as a surrogate for clinical events. Clinical care should integrate weight, diuretic response, residual congestion, perfusion, and renal electrolyte safety. Future trials should adopt a minimum reporting set comprising fixed-time weight, early urine output and urinary sodium, a standardized congestion score, discharge residual congestion, renal electrolyte safety, and ≥90-day death and heart failure events, enabling subsequent individual participant and trial-level validation of multidimensional profiles.

## Data Availability

The original contributions presented in the study are included in the article/supplementary material; further inquiries can be directed to the corresponding author.

## Appendix 1 Full electronic search strategies.

**Table:**

Source	Last searched	Executed search
PubMed/MEDLINE	18 July 2026	(“Heart Failure”[Mesh] OR “acute heart failure”[tiab] OR “acute decompensated heart failure”[tiab] OR “decompensated heart failure”[tiab] OR “worsening heart failure”[tiab]) AND (“Diuretics”[Mesh] OR “Ultrafiltration”[Mesh] OR decongest*[tiab] OR diure*[tiab] OR natriuresis[tiab] OR “urine sodium”[tiab] OR “urinary sodium”[tiab] OR “urine output”[tiab] OR “fluid loss”[tiab] OR “weight loss”[tiab] OR acetazolamide[tiab] OR thiazide*[tiab] OR tolvaptan[tiab] OR empagliflozin[tiab] OR dapagliflozin[tiab] OR ultrafiltration[tiab] OR “lung ultrasound”[tiab] OR “vena cava”[tiab]) AND (randomized controlled trial[pt] OR controlled clinical trial[pt] OR random*[tiab]) NOT (animals[mh] NOT humans[mh])
OpenAlex	18 July 2026	Five executed queries: “acute heart failure” decongestion randomized; “acute decompensated heart failure” diuretic randomized; “acute heart failure” natriuresis urine sodium randomized; “heart failure” ultrafiltration randomized; “acute heart failure” lung ultrasound decongestion randomized. Publication-date filter: January 1, 1990 to July 18, 2026; type: article.
ClinicalTrials.gov	18 July 2026	AREA[ConditionSearch](“Heart Failure”) AND AREA[StudyType](INTERVENTIONAL) AND (decongestion OR diuretic OR natriuresis OR “urine sodium” OR ultrafiltration OR acetazolamide OR thiazide OR tolvaptan OR empagliflozin OR dapagliflozin OR “lung ultrasound”)

## References

[1] McDonagh TA Metra M Adamo M Gardner RS Baumbach A Böhm M. 2021 ESC guidelines for the diagnosis and treatment of acute and chronic heart failure. Eur Heart J. (2021) 42(36):3599–726. 10.1093/eurheartj/ehab368 34447992

[2] Felker GM Lee KL Bull DA Redfield MM Stevenson LW Goldsmith SR. Diuretic strategies in patients with acute decompensated heart failure. N Engl J Med. (2011) 364(9):797–805. 10.1056/NEJMoa1005419 21366472 PMC3412356

[3] Kociol RD McNulty SE Hernandez AF Lee KL Redfield MM Tracy RP. Markers of decongestion, dyspnea relief, and clinical outcomes among patients hospitalized with acute heart failure. Circ Heart Fail. (2013) 6(2):240–5. 10.1161/CIRCHEARTFAILURE.112.969246 23250981 PMC3865520

[4] Gheorghiade M Konstam MA Burnett JC Grinfeld L Maggioni AP Swedberg K. Short-term clinical effects of tolvaptan, an oral vasopressin antagonist, in patients hospitalized for heart failure: the EVEREST clinical status trials. JAMA. (2007) 297(12):1332–43. 10.1001/jama.297.12.1332 17384438

[5] Konstam MA Gheorghiade M Burnett JC Grinfeld L Maggioni AP Swedberg K. Effects of oral tolvaptan in patients hospitalized for worsening heart failure: the EVEREST outcome trial. JAMA. (2007) 297(12):1319–31. 10.1001/jama.297.12.1319 17384437

[6] Costanzo MR Guglin ME Saltzberg MT Jessup ML Bart BA Teerlink JR. Ultrafiltration versus intravenous diuretics for patients hospitalized for acute decompensated heart failure. J Am Coll Cardiol. (2007) 49(6):675–83. 10.1016/j.jacc.2006.07.073 17291932

[7] Bart BA Goldsmith SR Lee KL Givertz MM O'Connor CM Bull DA. Ultrafiltration in decompensated heart failure with cardiorenal syndrome. N Engl J Med. (2012) 367(24):2296–304. 10.1056/NEJMoa1210357 23131078 PMC3690472

[8] Cannatà A Anastasia G De Marzo V Caspi O Bromage D Porto I. Diuretic strategies in acute heart failure: a systematic review and network meta-analysis of randomized clinical trials. Eur Heart J Cardiovasc Pharmacother. (2026) 12(1):6–14. 10.1093/ehjcvp/pvaf067 PMC12862974 40929381

[9] Abuelazm M Rakab MS Altamimi I Amin AM Rezq H Taha HI. Natriuresis-guided decongestion in acute decompensated heart failure: a systematic review and meta-analysis. J Cardiol. (2026) 87(6):502–12. 10.1016/j.jjcc.2025.12.013 41468992

[10] Page MJ McKenzie JE Bossuyt PM Boutron I Hoffmann TC Mulrow CD. The PRISMA 2020 statement: an updated guideline for reporting systematic reviews. Int J Surg. (2021) 88:105906. 10.1016/j.ijsu.2021.105906 33789826

[11] ClinicalTrials.gov. Effectiveness of ultrafiltration in treating people with acute decompensated heart failure and cardiorenal syndrome (The CARRESS Study). NCT00608491. Study results. Available online at: https://clinicaltrials.gov/study/NCT00608491 (Accessed October 26, 2026).

[12] ClinicalTrials.gov. Study of high-dose spironolactone vs. placebo therapy in acute heart failure. NCT02235077. Study results. Available online at: https://clinicaltrials.gov/study/NCT02235077 (Accessed October 26, 2026).

[13] ClinicalTrials.gov. Targeting acute congestion with tolvaptan in congestive heart failure. NCT01644331. Study results. Available online at: https://clinicaltrials.gov/study/NCT01644331 (Accessed October 26, 2026).

[14] Konstam MA Kiernan M Chandler A Dhingra R Mody FV Eisen H. Short-term effects of tolvaptan in patients with acute heart failure and volume overload. J Am Coll Cardiol. (2017) 69(11):1409–19. 10.1016/j.jacc.2016.12.035 28302292

[15] Sterne JAC Savović J Page MJ Elbers RG Blencowe NS Boutron I. Rob 2: a revised tool for assessing risk of bias in randomised trials. Br Med J. (2019) 366:l4898. 10.1136/bmj.l4898 31462531

[16] Buyse M Molenberghs G Burzykowski T Renard D Geys H. The validation of surrogate endpoints in meta-analyses of randomized experiments. Biostatistics. (2000) 1(1):49–67. 10.1093/biostatistics/1.1.49 12933525

[17] Daniels MJ Hughes MD. Meta-analysis for the evaluation of potential surrogate markers. Stat Med. (1997) 16(17):1965–82. 10.1002/(sici)1097-0258(19970915)16:17<1965::aid-sim630>3.0.co;2-m 9304767 10.1002/(sici)1097-0258(19970915)16:17<1965::aid-sim630>3.0.co;2-m

[18] Chen HH Anstrom KJ Givertz MM Stevenson LW Semigran MJ Goldsmith SR. Low-dose dopamine or low-dose nesiritide in acute heart failure with renal dysfunction: the ROSE acute heart failure randomized trial. JAMA. (2013) 310(23):2533–43. 10.1001/jama.2013.282190 24247300 PMC3934929

[19] Palazzuoli A Pellegrini M Ruocco G Martini G Franci B Campagna MS. Continuous versus bolus intermittent loop diuretic infusion in acutely decompensated heart failure: a prospective randomized trial. Crit Care. (2014) 18(3):R134. 10.1186/cc13952 24974232 PMC4227080

[20] Triposkiadis FK Butler J Karayannis G Starling RC Filippatos G Wolski K. Efficacy and safety of high dose versus low dose furosemide with or without dopamine infusion: the dopamine in acute decompensated heart failure II (DAD-HF II) trial. Int J Cardiol. (2014) 172(1):115–21. 10.1016/j.ijcard.2013.12.276 24485633

[21] Costanzo MR Negoianu D Jaski BE Bart BA Heywood JT Anand IS. Aquapheresis versus intravenous diuretics and hospitalizations for heart failure. JACC Heart Fail. (2016) 4(2):95–105. 10.1016/j.jchf.2015.08.005 26519995

[22] Felker GM Mentz RJ Cole RT Adams KF Egnaczyk GF Fiuzat M. Efficacy and safety of tolvaptan in patients hospitalized with acute heart failure. J Am Coll Cardiol. (2017) 69(11):1399–406. 10.1016/j.jacc.2016.09.004 27654854

[23] Butler J Anstrom KJ Felker GM Givertz MM Kalogeropoulos AP Konstam MA. Efficacy and safety of spironolactone in acute heart failure: the ATHENA-HF randomized clinical trial. JAMA Cardiol. (2017) 2(9):950–8. 10.1001/jamacardio.2017.2198 28700781 PMC5675712

[24] Cox ZL Hung R Lenihan DJ Testani JM. Diuretic strategies for loop diuretic resistance in acute heart failure: the 3T trial. JACC Heart Fail. (2020) 8(3):157–68. 10.1016/j.jchf.2019.09.012 31838029 PMC7058489

[25] Damman K Beusekamp JC Boorsma EM Swart HP Smilde TDJ Elvan A. Randomized, double-blind, placebo-controlled, multicentre pilot study on the effects of empagliflozin on clinical outcomes in patients with acute decompensated heart failure (EMPA-RESPONSE-AHF). Eur J Heart Fail. (2020) 22(4):713–22. 10.1002/ejhf.1713 31912605

[26] Hu J Wan Q Zhang Y Zhou J Li M Jiang L. Efficacy and safety of early ultrafiltration in patients with acute decompensated heart failure with volume overload: a prospective, randomized, controlled clinical trial. BMC Cardiovasc Disord. (2020) 20(1):447. 10.1186/s12872-020-01733-5 PMC7556949 33054727

[27] Schulze PC Bogoviku J Westphal J Aftanski P Haertel F Grund S. Effects of early empagliflozin initiation on diuresis and kidney function in patients with acute decompensated heart failure (EMPAG-HF). Circulation. (2022) 146(4):289–98. 10.1161/CIRCULATIONAHA.122.059038 35766022

[28] Voors AA Angermann CE Teerlink JR Collins SP Kosiborod M Biegus J. The SGLT2 inhibitor empagliflozin in patients hospitalized for acute heart failure: a multinational randomized trial. Nat Med. (2022) 28(3):568–74. 10.1038/s41591-021-01659-1 35228754 PMC8938265

[29] Biegus J Voors AA Collins SP Kosiborod MN Teerlink JR Angermann CE. Impact of empagliflozin on decongestion in acute heart failure: the EMPULSE trial. Eur Heart J. (2023) 44(1):41–50. 10.1093/eurheartj/ehac530 36254693 PMC9805406

[30] Mullens W Dauw J Martens P Verbrugge FH Nijst P Meekers E. Acetazolamide in acute decompensated heart failure with volume overload. N Engl J Med. (2022) 387(13):1185–95. 10.1056/NEJMoa2203094 36027559

[31] Trullàs JC Morales-Rull JL Casado J Carrera-Izquierdo M Sánchez-Marteles M Conde-Martel A. Combining loop with thiazide diuretics for decompensated heart failure: the CLOROTIC trial. Eur Heart J. (2023) 44(5):411–21. 10.1093/eurheartj/ehac689 36423214

[32] ter Maaten JM Beldhuis IE Van Der Meer P Krikken JA Postmus D Coster JE. Natriuresis-guided diuretic therapy in acute heart failure: a pragmatic randomized trial. Nat Med. (2023) 29(10):2625–32. 10.1038/s41591-023-02532-z 37640861 PMC10579092

[33] Emara AN Wadie M Mansour NO Shams MEE. The clinical outcomes of dapagliflozin in patients with acute heart failure: a randomized controlled trial (DAPA-RESPONSE-AHF). Eur J Pharmacol. (2023) 961:176179. 10.1016/j.ejphar.2023.176179 37923161

[34] Cox ZL Collins SP Hernandez GA McRae AT Davidson BT Adams K. Efficacy and safety of dapagliflozin in patients with acute heart failure. J Am Coll Cardiol. (2024) 83(14):1295–306. 10.1016/j.jacc.2024.02.009 38569758

[35] Burgos LM Baro Vila RC Ballari FN Goyeneche A Costabel JP Muñoz F. Inferior vena CAVA and lung ultrasound-guided therapy in acute heart failure: a randomized pilot study (CAVAL US-AHF study). Am Heart J. (2024) 277:47–57. 10.1016/j.ahj.2024.07.015 39094839

[36] Khorramshahi Bayat M Chan W Hay K McKenzie S Adhikari P Fincher G. Spot urinary sodium-guided titration of intravenous diuretic therapy in acute heart failure: a pilot randomized controlled trial. Eur Heart J Qual Care Clin Outcomes. (2025) 11(1):97–104. 10.1093/ehjqcco/qcae028 38632053

[37] Udelson JE Goldsmith SR Burkhoff D Colorado P Dinh W Gustafsson F. A multicentre, randomized, double-blind, active and placebo-controlled study of pecavaptan, a dual V1a/V2 vasopressin receptor antagonist, in patients with acute heart failure: the AVANTI trial. Eur J Heart Fail. (2025) 27(11):2525–36. 10.1002/ejhf.3801 40788619

[38] Jobs A Rausch TK König IR Vonthein R Devendra A Schäfer J. Inferior vena cava ultrasound to guide decongestion in acute decompensated heart failure: a randomized controlled trial. JACC Heart Fail. (2025) 13(10):102578. 10.1016/j.jchf.2025.102578 40929999

